# A pedigree-based cohort to study the genetic risk factors for cardiometabolic diseases: study design, baseline characteristics and preliminary results

**DOI:** 10.3389/fpubh.2023.1189993

**Published:** 2023-07-13

**Authors:** Hongchen Zheng, Ying Ye, Hui Huang, Chunlan Huang, Wenjing Gao, Mengying Wang, Wenyong Li, Ren Zhou, Jin Jiang, Siyue Wang, Canqing Yu, Jun Lv, Xiaoling Wu, Xiaoming Huang, Weihua Cao, Yansheng Yan, Kuicheng Zheng, Tao Wu, Liming Li

**Affiliations:** ^1^Department of Epidemiology and Biostatistics, School of Public Health, Peking University, Beijing, China; ^2^Key Laboratory of Epidemiology of Major Diseases (Peking University), Ministry of Education, Beijing, China; ^3^Key Laboratory of Carcinogenesis and Translational Research (Ministry of Education/Beijing), Laboratory of Genetics, Peking University Cancer Hospital and Institute, Beijing, China; ^4^Department of Local Diseases Control and Prevention, Fujian Provincial Center for Disease Control and Prevention, Fuzhou, China; ^5^Department of Hygiene, Nanjing Country Center for Disease Control and Prevention, Nanjing, China; ^6^Center for Public Health and Epidemic Preparedness and Response, Peking University, Beijing, China; ^7^Fujian Provincial Center for Disease Control and Prevention, Fuzhou, China; ^8^Key Laboratory of Reproductive Health, Ministry of Health, Beijing, China

**Keywords:** pedigree-based study, cohort study, baseline characteristic, heritability, cardiometabolic disease

## Abstract

**Background:**

We initiated the Fujian Tulou Pedigree-based Cohort (FTPC) as the integration of extended pedigrees and prospective cohort to clarify the genetic and environmental risk factors of cardiometabolic diseases.

**Methods:**

FTPC was carried out in Nanjing County, Fujian Province, China from August 2015 to December 2017 to recruit probands with the same surnames and then enroll their first-degree and more distant relatives. The participants were asked to complete questionnaire interview, physical examination, and blood collection. According to the local genealogical booklets and family registry, we reconstructed extended pedigrees to estimate the heritability of cardiometabolic traits. The follow-up of FTPC is scheduled every 5 years in the future.

**Results:**

The baseline survey interviewed 2,727 individuals in two clans. A total of 1,563 adult subjects who completed all baseline examinations were used to reconstruct pedigrees and 452 extended pedigrees were finally identified, including one seven-generation pedigree, two five-generation pedigrees, 23 four-generation pedigrees, 186 three-generation pedigrees, and 240 two-generation pedigrees. The average age of the participants was 57.4 years, with 43.6% being males. The prevalence of hypertension, diabetes and dyslipidemia in FTPC were 49.2, 10.0, and 45.2%, respectively. Based on the pedigree structure, the heritability of systolic blood pressure, diastolic blood pressure, fast blood glucose, total cholesterol, triglyceride, high-density lipoprotein, and low-density lipoprotein was estimated at 0.379, 0.306, 0.386, 0.452, 0.568, 0.852, and 0.387, respectively.

**Conclusion:**

As an extended pedigree cohort in China, FTPC will provide an important source to study both genetic and environmental risk factors prospectively.

## 1. Introduction

It is undisputed that cardiometabolic diseases are the predominant challenges to global health (1). In China, rapid environmental and economic changes accompanied by urbanization have led to the increasing prevalence of cardiometabolic diseases, including hypertension, heart diseases, diabetes, and stroke (2, 3). Despite the identification of a number of modifiable environmental risk factors, such as smoking, diet, and physical exercise, the accumulated knowledge and interventions targeting these risk factors have not yielded a significant reduction in the disease burden associated with cardiometabolic diseases (4). Therefore, the etiology of cardiometabolic diseases needs to be further explored. The insight into the genetics behind cardiometabolic diseases and their interaction with environmental factors may improve our understanding of cardiometabolic diseases and promote public health.

Prospective cohort studies provide a valuable advantage by allowing for the clarification of the temporal relationship between exposure and outcome in etiological studies. In contrast, traditional extended pedigree studies have been widely utilized to identify genetic determinants, offering improved control over population stratification in comparison to case–control designs (5, 6). Moreover, compared to twin or nuclear families, extended pedigrees have unique advantages in that they have various family relationships and can be applied to familial aggregation analysis, segregation analysis, linkage study, genetic association study, and estimation of heritability (7, 8). Pedigree-based cohort studies are designed to integrate pedigree studies and prospective cohorts to allow investigation of both genes and environment, separately or together (9). Follow-up of the pedigrees will also allow genetic and environmental risk factors to be studied prospectively.

Fujian Tulous represent a distinct style of rural dwellings found exclusively among the Hakka populations residing in the mountainous regions of southeastern Fujian province, China. These Tulous are characterized by their large, enclosed, and fortified earth buildings, typically exhibiting a rectangular or circular configuration. Notably, these structures feature remarkably thick load-bearing rammed earth walls, spanning three to five stories in height, capable of accommodating up to approximately 800 individuals (10). Typically, Fujian Tulous serve as the house for a single extended pedigree comprising multiple generations. As the clan expands, Tulou exhibits radial expansion by adding outer concentric rings or constructing additional earth buildings within close proximity, forming a cluster (10). Consequently, this architecture of Tulou facilitates convenient multi-generational investigations for genetic epidemiological studies. Owing to earth buildings’ unique architectural forms, special Hakka culture, and remote geographical location, native residents in southeastern Fujian province are isolated from other ethnic populations, resulting in their homogenous genetic backgrounds. Besides that, there is a periodic migration of workers and overseas nationals in the Tulou area. Therefore, the Tulou area provides a natural sample source for classical genetic epidemiological studies like pedigree studies and migrant studies.

Herein, we initiated Fujian Tulou Pedigree-based Cohort (FTPC) to explore the genetic and environmental risk factors for cardiometabolic diseases. In this manuscript, we described the study design, enrollment methods, collection of research data, and baseline characteristics of FTPC. We also presented a heritability estimation of cardiometabolic traits based on the pedigrees.

## 2. Methods

### 2.1. Study design

FTPC was an extended-pedigree-based prospective open cohort in the Tulou area of Nanjing County, Fujian Province. This study was designed to recruit any accessible multi-generation pedigrees. Probands were recruited according to their surname and then their first-degree and more distant relatives were invited to participate. A household was considered as a unit to be investigated and followed up prospectively.

### 2.2. Baseline investigation

The study sites were carefully selected based on clan clustering, patterns of major cardiometabolic diseases and exposures, the stability of the population, and long-term local commitment to the project. Ultimately, six villages were selected for participant recruitment. Participants with surname Zhang were enrolled from Taxia Village, Qujiang Village, and Nanou Village. And participants with surname Chen were enrolled from Caoban Village, Tumei Village, and Beiling Village. About half of the local residents were enrolled in our study. From August 2015 to December 2017, the baseline investigation was conducted to enroll all eligible participants and their relatives in the six villages. For migrant workers or overseas nationals, a complementary investigation was carried out when they returned home during Spring Festivals or Tomb-sweeping Days. Adults over 18 years old were asked to complete a face-to-face questionnaire and a family registry. Participants were also asked to conduct a physical examination and provide a blood sample for long-term storage. At the end of each survey day, a blood sub-sample was sent to the local hospital laboratory to conduct the biochemical examination. Those under 18 years old were only required to provide basic demographic information by their guardians. The study was approved by the Ethics Committee of Peking University Health Science Center. Written informed consent was obtained from all participants.

The inclusion criteria of the participants were as follows: (1) residents with surname Zhang or surname Chen who have accessible biological relatives and could provide complete genealogical information; (2) spouses of the probands; (3) volunteered to participate in the project and signed informed consent. Subjects with disabilities or other serious diseases were excluded if they were unable to complete the investigation.

### 2.3. Pedigree reconstruction

According to the genealogical booklets and on-site investigations, the family registry collected information about the participants and their relatives’ names, kinships, and dates of birth. Trained investigators were asked to interview relatives of the probands as many as possible. According to the family registration, pedigrees were reconstructed using R version 3.6.2.

### 2.4. Data collection and outcome definition

The questionnaire interview included the following information: (1) demographic information; (2) social-economic status (educational level, occupation, marriage status, and annual income); (3) health-related behaviors (smoking, alcohol consumption, tea consumption, diet, and physical activity); (4) health status (disease history and medication history); (5) menstrual and obstetrical histories for women (menstrual cycle, menopausal age, pregnancy history, and birth history); (6) psychological status assessments (adapted Kessler10) (11).

Physical examinations included: (1) height; (2) body weight and body fat percentage; (3) waist circumference and hip circumference; (4) blood pressure and heart rate. These examinations were conducted twice per subject. If the difference between the two blood pressure measures was greater than 10 mmHg, a third measure would be conducted. The average of the last two measurements was recorded and used in the analysis.

For each participant, a 15 mL fasting blood sample was collected to conduct biochemical examinations (2 mL EDTA anticoagulant sample) and future genotyping (8 mL EDTA anticoagulant sample and 5 mL coagulant sample). Biochemical examinations included: (1) blood lipids levels such as total cholesterol, triglyceride, high-density lipoprotein, and low-density lipoprotein; (2) liver function markers such as aspartate aminotransferase, alanine aminotransferase, glutamyl transpeptidase, total protein, albumin, globulin, and total bilirubin; (3) renal function markers such as uric acid, creatinine, and uric acid nitrogen; (4) fasting blood glucose.

Hypertension was defined if the participant was under any following conditions: (1) self-reported hypertension diagnosed by secondary or higher-level hospitals; (2) taking antihypertension drugs; (3) systolic blood pressure (SBP) ≥ 140 mmHg and/or diastolic blood pressure (DBP) ≥ 90 mmHg (12). Diabetes was defined if the participant was under any following conditions: (1) self-reported diabetes diagnosed by secondary or higher-level hospitals; (2) fasting blood glucose ≥7.0 mmol/L. Dyslipidemia was defined if the participant was under any following conditions: (1) self-reported hyperlipidemia diagnosed by secondary or higher-level hospitals; (2) total cholesterol ≥6.22 mmol/L and/or triglyceride ≥2.26 mmol/L and/or high-density lipoprotein ≤1.04 mmol/L and/or low-density lipoprotein ≥4.14 mmol/L. (13) Awareness rate referred to the proportion of the number of self-reported cases to the total number of participants with specific diseases. Treatment rate referred to the proportion of the number of cases under treatment to the total number of participants with specific diseases.

### 2.5. Long-term follow-up

The active follow-up interview is scheduled at a 5-year interval among all surviving participants. The information about any incident cases of stroke, coronary heart disease, cancer, and other non-communicable diseases will be collected through a passive linkage with records of local health surveillance system. The death events will be obtained from the Death Registry of the National Centers for Disease Control and Prevention. All participants will be followed up indefinitely until cause-specific mortality is observed. The first wave of follow-up has initiated on April 2023.

### 2.6. Quality control

FTPC adopted a unified manual for field investigation and standard criteria for the physical and biochemical examination. The collected data was checked regularly during the study period by the study coordinating centers. Most questionnaires were electronically collected with a logical verification to avoid systematic errors.

### 2.7. Descriptive analysis and heritability estimation

In this study, baseline characteristics of participants in FTPC were present with means (medians for inormally distributed variables) and proportions for the continuous and categorical variables, respectively. Descriptive analysis was conducted in Stata version 13.1.

The maximum likelihood-based variance components decomposition and liability-threshold model were used to estimate the heritability of continuous and categorical variables, respectively (14, 15). The estimation of heritability was conducted in Solar version 8.1.1 after adjusting for age, sex, age^2^, age by sex, and age^2^ by sex. Lipid and blood pressure values were corrected for the use of lipid-lowering and antihypertensive medication using published constants (total cholesterol +1.347 mmoL/L, triglyceride +0.208 mmoL/L, high-density lipoprotein −0.060 mmoL/L, low-density lipoprotein +1.290 mmoL/L, SBP +15 mmHg, DBP +10 mmHg) (16–18). *p* values <0.05 were defined as statistically significant.

## 3. Results

### 3.1. Baseline characteristics

From August 2015 to December 2017, 2,727 individuals were interviewed in clan Zhang and clan Chen (Supplementary Figure S1). As shown in Table 1, a total of 1,563 adult subjects who completed the baseline survey including questionnaire interviews, physical examinations, blood sample collection, and biochemical examinations were included in the pedigree reconstruction. The average age of the participants was 57.2 years and males accounted for 43.6%. A majority of the participants in FTPC tended to have a low level of education, a low level of income, a habit of tea consumption every day, and a preference for light food. Results of physical and biochemical examinations were shown in Supplementary Table S1. The participants were short in stature with abdominal obesity and the average height and weight were 158.5 cm and 58.9 kg, respectively. In addition, the average SBP and serum lipid values were close to the diagnostic criteria of the diseases, suggesting a high risk of hypertension and dyslipidemia for the participants in FTPC.

**Table 1:** Baseline characteristics of the participants for Fujian Tulou Pedigree-based Cohort.

Variables	Males		Females		Total	
	Number	%	Number	%	Number	%
Gender	681	43.6	882	56.4	1,563	100.0
Age (median (quartile))	59 (50–67)		56 (48–65)		58 (49–66)	
Marriage status						
Married	632	92.8	768	87.1	1,400	89.6
Divorce	29	4.3	96	10.9	125	8.0
Unmarried	20	2.9	18	2.0	38	2.4
Education level						
Lower than primary	115	16.9	417	47.3	532	34.0
Primary	221	32.5	222	25.2	443	28.3
Junior	222	32.6	149	16.9	371	23.7
High	101	14.8	66	7.5	167	10.7
College or above	22	3.2	28	3.2	50	3.2
Occupation						
Farmer or worker	371	54.5	368	41.7	739	47.3
Administrative manager or professional worker	30	4.4	28	3.2	58	3.7
Pensioner or student	76	11.2	73	8.3	149	9.5
Others	204	30.0	413	46.8	617	39.5
Annual income						
<10 k RMB	140	20.6	194	22.0	334	21.4
10-50 k RMB	369	54.2	495	56.1	864	55.3
>50 k RMB	172	25.3	193	21.9	365	23.4
Smoking status						
Current smoking	283	41.6	22	2.5	305	19.5
Ever-smoking	94	13.8	3	0.3	97	6.2
Never smoking	304	44.6	857	97.2	1,161	74.3
Alcohol consumption						
Current drinking	137	20.1	29	3.3	166	10.6
Ever-drinking	31	4.6	6	0.7	37	2.4
Never drinking	513	75.3	847	96.0	1,360	87.0
Drinking tea						
Less than 1 day per week	221	32.5	584	66.2	805	51.5
1–2 days per week	9	1.3	11	1.3	20	1.3
3–5 days per week	18	2.6	19	2.2	37	2.4
Almost every day	433	63.6	268	30.4	701	44.8
Spicy food consumption						
Never	480	70.5	665	75.4	1,145	73.3
Less than once per week	133	19.5	150	17.0	283	18.1
1–2 times per week	42	6.2	43	4.9	85	5.4
3–5 times per week	7	1.0	9	1.0	16	1.0
Almost every day	19	2.8	15	1.7	34	2.2

Based on physical and biochemical examinations, the prevalence of hypertension, diabetes, and dyslipidemia was estimated at 49.2, 10.0, and 45.2%, respectively, which were much higher than the self-reported prevalence (Table 2). Despite the heavy burden of these diseases, over half of the patients were unaware of their disease status and received no treatment.

**Table 2:** The burden of hypertension, dyslipidemia, and diabetes for Fujian Tulou Pedigree-based Cohort.

Disease	Self-reported		Investigation results			
	Number of cases	Prevalence, %	Number of cases	Prevalence, %	Awareness rate, %	Treatment rate, %
Hypertension	226	14.5	769	49.2	29.4	23.9
Dyslipidemia	17	1.1	706	45.2	2.4	2.4
Diabetes	64	4.1	157	10.0	40.8	39.5

### 3.2. Pedigree reconstruction

Based on the pedigree registry, 452 pedigrees were reconstructed, including one seven-generation pedigree, two five-generation pedigrees, 23 four-generation pedigrees, 186 three-generation pedigrees, and 240 two-generation pedigrees (Table 3). More than half of the family members completed the baseline investigation and an average of 3.46 family members were investigated per pedigree. There were rich family relationships extracted from the pedigrees (Table 4) and the majority of the family relationships were parent-offspring and siblings. The family tree of the seven-generation pedigree was displayed in Supplementary Figure S2.

**Table 3:** Pedigree composition and investigation proportion of Fujian Tulou Pedigree-based Cohort.

Number of generations	Number of pedigrees	Total number of family members	Number of family members investigated	Average number of family members investigated	Investigation proportion, %
2	240	751	503	2.10	70.0
3	186	1,575	814	4.38	51.7
4	23	380	191	8.30	50.3
5	2	50	23	11.50	46.0
7	1	62	32	32.00	51.6
Total	452	2,818	1,563	3.46	55.5

Average number of family members investigated = number of family members investigated/number of pedigrees. Investigation proportion = number of family members investigated/total number of family members.

**Table 4:** Family relationships extracted from Fujian Tulou Pedigree-based Cohort.

Relationship	Number of pairs
Parent-offspring	338
Siblings	413
Grandparent-grandchild	33
Uncle/aunt-nephew	128
Third degree	19
Forth degree	5
Fifth degree	13
Sixth degree	6
Seventh degree	6

### 3.3. Heritability estimation

Based on the pedigree structure, we generated the heritability estimations for cardiometabolic traits (Table 5). The heritability of SBP, DBP, and hypertension was estimated at a similar level of 0.387 (95% confidence interval (CI), 0.219–0.554), 0.365 (95% CI, 0.201–0.529), and 0.404 (95% CI, 0.134–0.671), respectively. For fast blood glucose and diabetes, the heritability was estimated at 0.386 (95% CI, 0.237–0.535) and 0.272 (95% CI, −0.166–0.710), respectively. In contrast, the heritability estimations of serum lipid values and dyslipidemia were much higher than that of blood glucose or blood pressure. Heritability estimations for non-cardiometabolic traits were shown in Supplementary Table S2.

**Table 5:** Heritability estimation of cardiometabolic traits based on extended pedigrees.

Variables	Effect of covariates^*^	Heritability	95% confidence interval	*p* values
Systolic blood pressure^**^	0.215	0.387	0.219 to 0.554	<0.01
Diastolic blood pressure^**^	0.078	0.365	0.201 to 0.529	<0.01
Hypertension	–	0.404	0.134 to 0.671	<0.01
Fast blood glucose^**^	0.047	0.386	0.237 to 0.535	<0.01
Diabetes	–	0.272	−0.166 to 0.710	0.11
Total cholesterol^**^	0.034	0.452	0.306 to 0.598	<0.01
Triglyceride^**^	0.040	0.568	0.429 to 0.707	<0.01
High-density lipoprotein^**^	0.037	0.852	0.741 to 0.964	<0.01
Low-density lipoprotein	0.042	0.387	0.236 to 0.535	<0.01
Dyslipidemia	–	0.799	0.592 to 1.006	<0.01

^*^Adjusted for age, sex, age^2^, age by sex, and age^2^ by sex. ^**^Traits are non-normally distributed and inverse normalized transformation is applied for heritability estimation.

## 4. Discussion

The rapidly rising prevalence of cardiometabolic diseases has made them the predominant disease burden in China, with the prevalence of hypertension alone increasing 2.5 times from 2002 to 2017 (19, 20). However, the identification of risk factors for cardiometabolic diseases and intervention measurements do not bring in a significant reduction in the disease burden. These facts underscore the significance of gaining a comprehensive understanding of genetic determinants. By exploring potential gene–environment interactions, valuable insights can be gained to facilitate population stratification for targeted behavior interventions. FTPC is thus designed as an integration of extended pedigrees and prospective cohort to explore the genetic and environmental risk factors together. The pedigree study is a classical design for genetic epidemiological studies because the pedigrees will minimize the bias caused by population stratification, especially when involving participants from different cultural and ethnic background (21). Extended pedigrees have natural advantages in various intergenerational and intragenerational relationships to allow linkage studies and association studies for common and rare mutations (7, 22, 23). In contrast to the case–control studies enrolling unrelated individuals, extended pedigrees could provide higher statistical power to detect gene–environment interactions with the same number of disease cases (24–26). When following up the extended pedigrees prospectively, we could further study the effect of gene, environment, and gene–environment interactions on disease incidents. In this context, we initiated FTPC, an extended-pedigree-based cohort study, to explore the genetic and environmental risk factors for cardiometabolic diseases.

We decided to establish our pedigree-based cohort in the Tulou area, which is an ideal region for recruiting extended pedigrees. As one Tulou is occupied by one large family clan, the family members generally live close and have homogeneous genetic backgrounds. Because of the remote location, traffic inconvenience, and native tradition, local residents in the Tulou area rarely move outside. Driven by the strong faith in clan culture, migrant workers always come back home and visit their relatives at regular intervals. These features make it possible to recruit more family members of a pedigree and follow them up for a long time. In this study, we successfully identified extended pedigrees of different family sizes and extracted various family relationships. These pedigrees will provide an important source to conduct any type of genetic epidemiological study. There is a unique Hakka culture in the Tulou area where local residents have distinctive living habits such as a preference for tea consumption and light food. The insight into the association between those living habits and cardiometabolic diseases may contribute to the understanding of modifiable risk factors and further intervention measurements. Besides that, by comparing the migrant workers and local residents, FTPC will provide the possibility to study the effect of migration and social determinants in the future.

FTPC is based in a typical rural region in southern China with a heavy burden of cardiometabolic diseases. We adopt a means of snowball sampling to recruit participants for FTPC where probands are first identified and their relatives are then investigated. Such a sampling method will naturally result in a higher prevalence of cardiometabolic diseases because of the familial aggregation for these diseases. According to the baseline investigation, the prevalence of hypertension, diabetes, and dyslipidemia in FTPC is reported to be 49.2, 10.0, and 45.2%, respectively, which is even higher than that in urban areas of China (19, 27, 28). The high prevalence of cardiometabolic diseases indicates a compelling need for further etiological studies to elucidate the underlying causes of these conditions. In contrast to the prevalence based on investigation results, the self-reported prevalence in FTPC is much lower. No more than 3% of dyslipidemia patients are aware of their disease status or receive medical treatment. This phenomenon of “high prevalence and low treatment rate” may be due to poor health awareness and exposure to risk factors among the local residents. It is thus of great potential to carry out studies on the effect of behavioral intervention and health education in the Tulou area.

Our cohort has begun to make significant contributions to the estimation of heritability for cardiometabolic traits. Based on the pedigree structure, we obtained heritability estimations for SBP, DBP, and hypertension, ranging from 0.36 to 0.40. This finding suggests that the genetic component may have a similar effect on the variation of blood pressure and liability of hypertension, or there may be the same susceptible genes to determine the blood pressure values and hypertension. The heritability for SBP and DBP in FTPC is consistent with the estimation in Nigerian families but lower than the estimation from the previous meta-analysis of twin studies (29–31). Compared with twin studies, extended pedigrees often generate conservative results because of various family relationships and fewer theoretical assumptions (32). For fast blood glucose, we get a heritability of 0.386 (95% CI, 0.237–0.535) but the heritability of diabetes is not statistically significant. This result suggests that in this population, genetic determinants may contribute to the variation of blood glucose values to some extent but may not affect the liability of diabetes. For serum lipid markers, their heritability is much higher than blood pressure and blood glucose, suggesting that there is a strong effect of genetic determinants on serum lipid values. Using the pedigrees in this study, we derived overall estimations of heritability for cardiometabolic traits, which supports the findings of prior genomic investigations. In future research, we can leverage the pedigrees to further explore the genetic correlations and gene–environment interactions among cardiometabolic traits at the phenotypic level. Supplementary Table S2 presents the heritability of non-cardiometabolic traits, which exhibit variations in magnitudes while generally converging around 0.5. These results imply that both genetic and environmental factors contribute to the manifestation of these traits, emphasizing the need for a more profound understanding of the genetic and environmental etiologies, as well as their interactions. Additionally, we also look forward to conducting multi-omics studies to estimate heritability and explore genetic risk loci with genotype data.

This study has some limitations. More than half of the pedigrees in FTPC are two-generation pedigrees because of inadequate genealogical information. To address this limitation and expand our pedigrees, we have incorporated an additional village into our ongoing investigation. Our aim is to gather more comprehensive family information, enabling the connection of two-generation pedigrees and the enrollment of additional members from multi-generation pedigrees.

Because of the difficulties in recruitment, investigation, and follow-up of the pedigrees, there are only several extended-pedigree-based studies in the world, such as Nigerian families and Oman families (29, 33). In China, other types of family-based studies have been carried out to explore genetic risk factors, for example, twin studies enrolling monozygotic and dizygotic twins, sibling studies enrolling pairs of siblings with different disease statuses, and nuclear family studies enrolling case-parent trios (34–37). In the present study, we initiated FTPC to enroll the extended pedigrees with multi-generations. This cohort provides a source for almost all types of family relationships and will be a supplementary resource for genetic epidemiological studies. As an extended-pedigree-based cohort in China, we believe that our cohort will play an important role in the identification of environmental and genetic risk factors of cardiometabolic diseases and other non-communicable diseases.

## Data availability statement

The datasets presented in this article are not readily available because Data used in this study are available from the corresponding authors upon reasonable request. Requests to access the datasets should be directed to TW, twu@bjmu.edu.cn.

## Ethics statement

The studies involving human participants were reviewed and approved by the Ethics Committee of Peking University Health Science Center. The patients/participants provided their written informed consent to participate in this study.

## Author contributions

All authors listed have made a substantial, direct, and intellectual contribution to the work and approved it for publication.

## Funding

This work was supported by Special Fund for Health Scientific Research of Public Welfare (201502006), Fujian Provincial Health Technology Project (2020CXB009), Natural Science Foundation of Fujian Province, China (2021 J01352), and Peking University Outstanding Discipline Construction Project of Epidemiology and Biostatistics.

## Conflict of interest

The authors declare that the research was conducted in the absence of any commercial or financial relationships that could be construed as a potential conflict of interest.

## Publisher’s note

All claims expressed in this article are solely those of the authors and do not necessarily represent those of their affiliated organizations, or those of the publisher, the editors and the reviewers. Any product that may be evaluated in this article, or claim that may be made by its manufacturer, is not guaranteed or endorsed by the publisher.
