# Supplementary material for: A pedigree-based cohort to study the genetic risk factors for cardiometabolic diseases: study design, baseline characteristics and preliminary results
Source: Front Public Health. 2023 Jul 13;11:1189993. doi: 10.3389/fpubh.2023.1189993 (PMC10374840; doi:10.3389/fpubh.2023.1189993)
Supplement: Supplementary file 1 [file Data_Sheet_2.PDF]

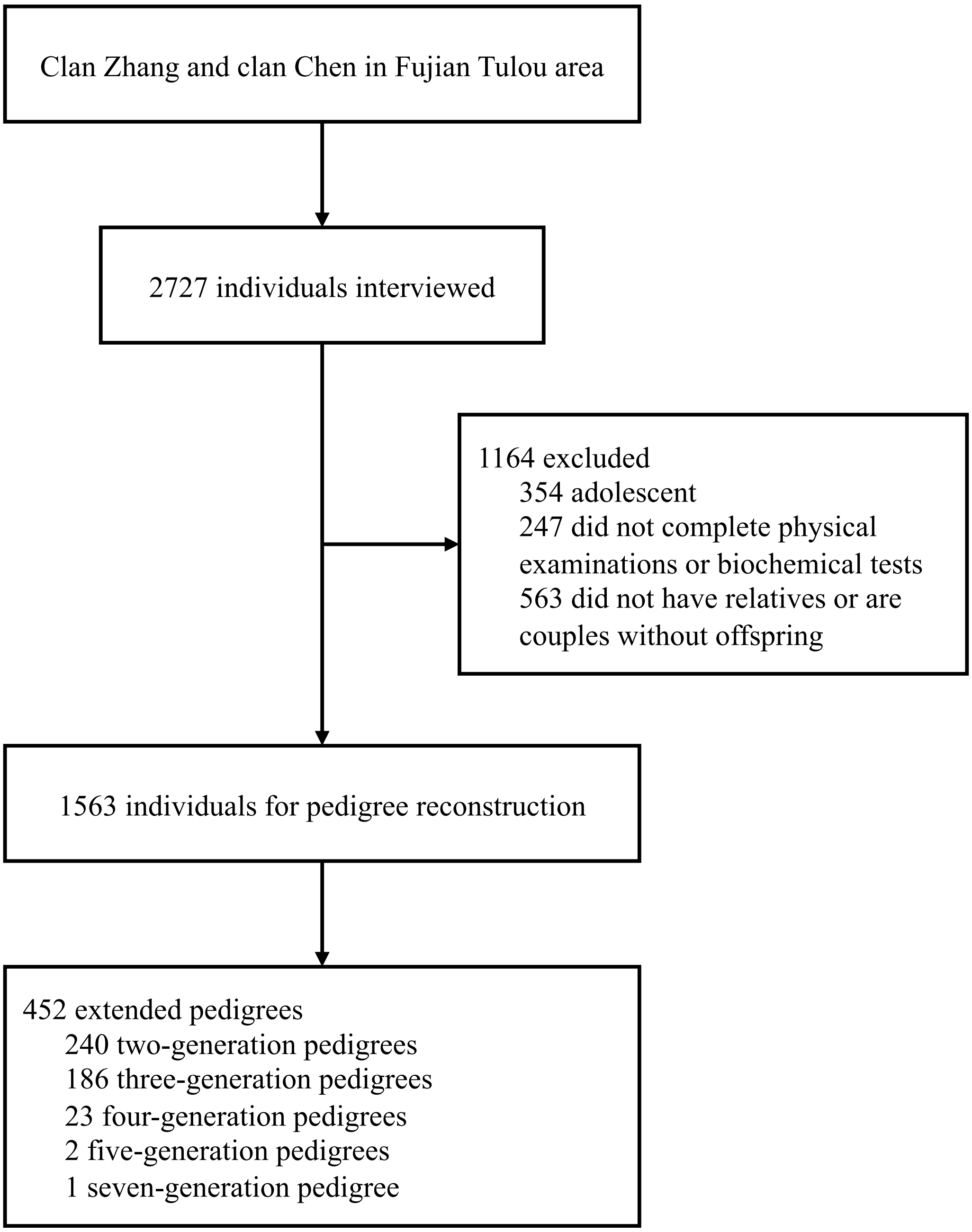


Supplementary Figure 1

The flow chart for participant enrollment and pedigree reconstruction

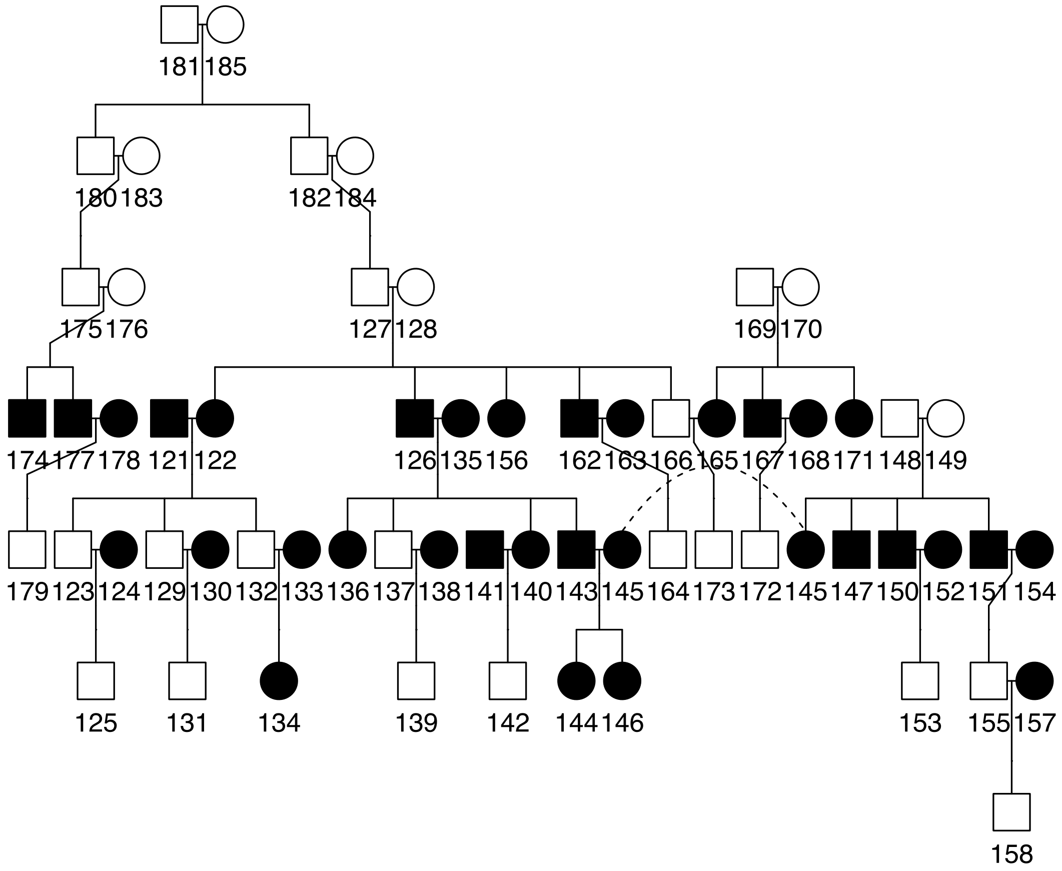


Supplementary Figure 2

The seven-generation pedigree in Fujian Tulou Pedigree-based Cohort

The hollow means he/she has not been investigated. The solid means he/she has been investigated. The square means male. The circle means female. Imaginary line means the same person.

Supplementary Table 1

Physical examinations and biochemical examinations of the participants for Fujian Tulou Pedigree-based Cohort

| Variables | Male (N=681) | Female (N=882) | Total (N=1563) | P value*** |
|---|---|---|---|---|
| Weight* (kg) | 63.8 (56.5 - 70.3) | 55.2 (48.9 - 60.4) | 58.9 (51.7 - 65.1) | <0.01 |
| Height** (cm) | 164.8 (6.4) | 153.7 (6.2) | 158.5 (8.3) | <0.01 |
| Waist circumference** (cm) | 85.5 (9.9) | 81.6 (9.4) | 83.3 (9.8) | <0.01 |
| Hip circumference** (cm) | 94.6 (7.6) | 93.7 (6.8) | 94.1 (7.2) | 0.01 |
| Body mass index** (kg/m$^2$) | 23.4 (3.5) | 23.3 (3.2) | 23.4 (3.3) | 0.55 |
| Symbolic blood pressure* (mmHg) | 139.6 (125.0 - 154.0) | 137.3 (120.5 - 154.0) | 138.3 (122.5 - 153.5) | 0.02 |
| Diabolic blood pressure* (mmHg) | 82.0 (75.0 - 89.5) | 76.5 (71.0 – 85.0) | 79.5 (72.0 – 87.0) | <0.01 |
| Heart rate* (times per min) | 78.3 (70.5 - 85.0) | 77.9 (71.0 - 84.0) | 78.1 (70.5 - 84.5) | 0.46 |
| Blood lipid marker | | | | |
| Total cholesterol* (mmol/L) | 4.8 (4.1 - 5.4) | 5.0 (4.3 - 5.9) | 5.0 (4.2 - 5.7) | <0.01 |
| Triglyceride* (mmol/L) | 1.9 (1.0 - 2.0) | 1.6 (0.9 - 2.0) | 1.8 (1.0 - 2.0) | <0.01 |
| High-density lipoprotein* (mmol/L) | 1.1 (0.9 - 1.5) | 1.4 (1.0 - 1.9) | 1.3 (1.0 - 1.7) | <0.01 |
| Low-density lipoprotein** (mmol/L) | 3.0 (0.8) | 3.1 (0.8) | 3.0 (0.8) | 0.03 |
| Liver function marker | | | | |
| Aspartate aminotransferase* (U/T) | 26.4 (21.0 - 28.0) | 24.4 (19.1 - 26.4) | 25.3 (19.9 - 27.1) | <0.01 |
| Alanine aminotransferase* (U/T) | 27.0 (18.0 - 31.1) | 23.0 (15.2 - 24.9) | 24.7 (16.1 - 27.9) | <0.01 |
| Glutamyl transpeptidase* (U/T) | 44.3 (22.5 - 46.7) | 27.9 (15.3 - 28.4) | 35.0 (17.7 - 36.4) | <0.01 |
| Total protein* (g/L) | 75.3 (71.5 - 76.9) | 75.7 (72.9 - 78.4) | 75.5 (72.0 - 78.0) | <0.01 |
| Albumin* (g/L) | 46.7 (45.0 - 48.2) | 46.1 (44.9 - 48.0) | 46.4 (44.9 - 48.0) | 0.60 |
| Globulin** (g/L) | 27.7 (3.7) | 29.3 (3.6) | 28.6 (3.7) | <0.01 |
| Total bilirubin** (umol/L) | 13.7 (7.1) | 12.0 (8.2) | 12.8 (7.8) | <0.01 |
| Fasting blood glucose* (mmol/L) | 5.5 (5.0 - 6.0) | 5.3 (5.0 - 6.0) | 5.4 (5.0 - 6.0) | <0.01 |
| Renal function marker | | | | |
| Uric acid** (umol/L) | 337.5 (84.2) | 267.0 (68.8) | 297.7 (83.5) | <0.01 |
| Creatinine** (umol/L) | 101.5 (12.9) | 84.7 (11.4) | 92.0 (14.7) | <0.01 |
| Uric acid nitrogen* (umol/L) | 5.9 (4.0 - 6.2) | 5.7 (3.8 - 5.9) | 5.8 (4.0 - 6.0) | 0.68 |

*Traits are non-normally distributed and values in the cells are medians (quartiles).

[**]Traits are normally distributed and values in the cells are means (standard deviation).

[***]P-values for the test of difference between genders

Supplementary Table 2

Heritability estimation of non-cardiometabolic traits based on extended pedigrees

| Variables | Effect of covariates* | Heritability | 95% confidence interval | P values |
|---|---|---|---|---|
| Weight** (kg) | 0.248 | 0.498 | 0.363 - 0.633 | <0.001 |
| Height (cm) | 0.518 | 0.612 | 0.466 - 0.758 | <0.001 |
| Waist circumference (cm) | 0.091 | 0.479 | 0.341 - 0.619 | <0.001 |
| Hip circumference (cm) | 0.021 | 0.364 | 0.220 - 0.507 | <0.001 |
| Body mass index (kg/m$^2$) | 0.057 | 0.419 | 0.276 - 0.561 | <0.001 |
| Heart rate** (times per min) | 0.022 | 0.263 | 0.120 - 0.407 | <0.001 |
| Liver function marker | | | | |
| Aspartate aminotransferase** (U/T) | 0.086 | 0.345 | 0.197 - 0.492 | 0.009 |
| Alanine aminotransferase** (U/T) | 0.093 | 0.359 | 0.211 - 0.506 | <0.001 |
| Glutamyl transpeptidase** (U/T) | 0.151 | 0.453 | 0.298 - 0.608 | <0.001 |
| Total protein** (g/L) | 0.031 | 0.276 | 0.114 - 0.438 | <0.001 |
| Albumin** (g/L) | 0.063 | 0.340 | 0.179 - 0.502 | <0.001 |
| Globulin (g/L) | 0.071 | 0.465 | 0.311 - 0.619 | <0.001 |
| Total bilirubin (umol/L) | 0.023 | 0.453 | 0.299 - 0.607 | <0.001 |
| Renal function marker | | | | |
| Uric acid (umol/L) | 0.209 | 0.635 | 0.485 - 0.786 | <0.001 |
| Creatinine (umol/L) | 0.362 | 0.298 | 0.122 - 0.473 | <0.001 |
| Uric acid nitrogen** (umol/L) | 0.037 | 0.631 | 0.501 - 0.761 | <0.001 |

*Adjusted for age, sex, age$^2$, age by sex, and age$^2$ by sex.

**Traits are non-normally distributed and inverse normalized transformation is applied for heritability estimation.
